# The Role of Transjugular Intrahepatic Portosystemic Shunt (TIPS) in Treating Portal Hypertension in Patients with Hepatocellular Carcinoma

**DOI:** 10.3390/medicina59061150

**Published:** 2023-06-15

**Authors:** Daniele Balducci, Michele Montori, Federico De Blasio, Alessandro Di Bucchianico, Maria Eva Argenziano, Gianluca Svegliati Baroni, Emidio Scarpellini

**Affiliations:** 1 Clinic of Gastroenterology, Hepatology and Emergency Digestive Endoscopy, Università Politecnica delle Marche, 60126 Ancona, Italy; 2 Liver Disease and Transplant Unit, Università Politecnica delle Marche, 60126 Ancona, Italy; 3 Clinical Nutrition Unit and Internal Medicine Unit, “Madonna del Soccorso” General Hospital, Via Luciano Manara 7, 63074 San Benedetto del Tronto, Italy; 4 Translational Research Center for Gastrointestinal Disease (T.A.R.G.I.D.), Gasthuisberg University Hospital, KULeuven, Herestraat 49, 3000 Lueven, Belgium

**Keywords:** transjugular intrahepatic portosystemic shunt, hepatocellular carcinoma, locoregional therapy, systemic therapy, liver surgery

## Abstract

Liver cancer is very frequent, and hepatocellular carcinoma (HCC) accounts for the majority of liver cancer cases. Its growing incidence has been greatly affected by the increasing prevalence of metabolic-associated fatty liver disease (MAFLD). The latter is a new epidemic in our era. In fact, HCC is often generated from noncirrhotic liver and its treatment benefits from surgical and nonsurgical approaches, potentially bridged by transjugular intrahepatic portosystemic shunt (TIPS) use. TIPS use is an effective treatment for portal hypertension complications, but its application in patients with HCC and clinically significant portal hypertension (CSPH) remains controversial due to concerns about tumor rupture, dissemination, and increased toxicity. The technical feasibility and safety of TIPS use in HCC patients have been evaluated in several studies. Despite concerns about intraprocedural complications, retrospective studies have shown high success rates and low complication rates in TIPS placement for HCC patients. TIPS use in combination with locoregional treatments, such as transarterial chemoembolization (TACE) or transarterial radioembolization (TARE), has been explored as a treatment option for HCC patients with portal hypertension. These studies have shown improved survival rates in patients undergoing TIPS in combination with locoregional treatments. However, the efficacy and toxicity of TACE in combination with TIPS use require careful evaluation, as changes in venous and arterial flow can affect treatment outcomes and complications. The results from studies evaluating the impact of TIPS on systemic therapy and surgical options are also promising. In conclusion, the TIPS is a sufficiently safe, useful item available for physicians treating complications of portal hypertension. Moreover, a TIPS can be used in combination with locoregional therapy in HCC patients. Systemic chemotherapy can also benefit of the use of TIPS placement. A complex interplay affects TIPS use with surgery. The latter needs further data. The TIPS is a useful and safe add-on treatment, changing the natural course of HCC progression. Its use is regulated by a sophisticated physiologic and pathophysiologic flow of evidence.

## 1. Introduction

**Figure 1:**
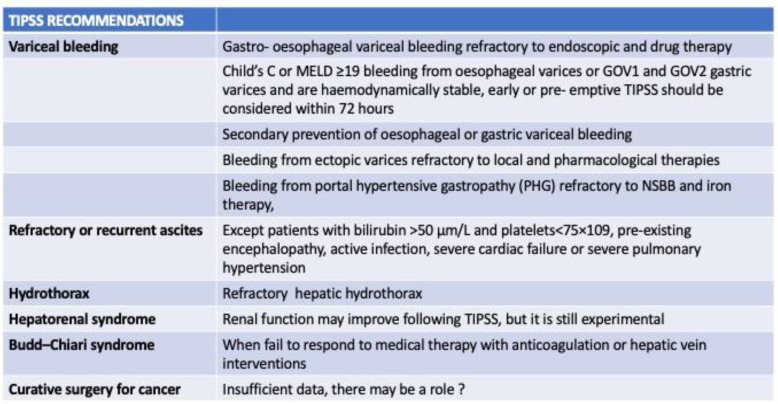
Summary of main TIPSS recommendations.

Liver cancer is the seventh most frequent cancer worldwide and the second most frequent cause of cancer-related mortality [1]. Hepatocellular carcinoma (HCC) accounts for about 75% of the total cases; interestingly, it is one of the main contributors to the world’s cancer burden [2]. The most important risk factors for HCC development are hepatitis B virus (HBV)/ hepatitis C virus (HCV) chronic infections, alcohol intake, and aflatoxin exposure [3]. However, increasing evidence supports the idea that metabolic syndrome, diabetes, and obesity may also play an important role in HCC development [4]. Moreover, the incidence of HCC is greatly increased in cirrhosis, regardless of the underlying etiology [5]. Transjugular intrahepatic portosystemic shunt (TIPS) is an effective treatment for portal hypertension complications. Typical indications for TIPS are refractory ascites and secondary prevention of variceal bleeding. Indeed, more recently, its application has also been used to treat acute refractory variceal bleeding, hepatorenal syndrome, and hepatic hydrothorax [6] (Figure 1). Although TIPS use can effectively reduce portal hypertension and may improve survival in some cirrhotic patients [7], to date, there is no clear consensus on the best treatment strategy in patients with HCC and clinically significant portal hypertension (CSPH). In fact, TIPS positioning in patients with HCC involves specific concerns: the possibility of tumor rapture and bleeding [8], tumor dissemination [9], increased toxicity, and complications of locoregional therapy [10]. On the other hand, TIPS use may play as critical role in some patients with CSPH, allowing the possibility of systemic therapy [11] and/or surgery [12,13]. 

Thus, in this article, we outline the possible role of TIPS use in patients with HCC in different scenarios.

## 2. Technical Feasibility and Safety of TIPS Use in HCC Patients

The presence of extensive hepatic malignancy is considered a relative contraindication to TIPS use. In addition, one of the factors limiting the application of TIPS placement in patients with portal hypertension and liver cancer is its feasibility and safety. The major concern linked to this procedure is the development of intraprocedural complications (e.g., liver failure, tumor rupture, or bleeding) [8]. However, in one of the largest retrospective studies accounting for 209 cases of TIPS placement in HCC patients, Qui et al. [14] showed that 97.69% of the TIPS procedures were successfully completed, with no serious procedure-related complications. Another issue may be linked to HCC dimensions: it was reported that large liver tumors are prone to rupture. Nevertheless, there was no evidence of an association of rupture with tumor size during TIPS placement in this enrolled cohort [14]. On the other hand, Liu et al. [15] reported tumor rupture in 8.6% of patients. Indeed, this was reported mainly in tumors bigger than 10 cm, and the cohort was significantly smaller than the previous one. In a retrospective study by Tsauo et al. [16], the overall technical success rate of the procedure in 126 cirrhotic patients was very high (98.4%). There were only two cases of major-procedure-related acute complications (namely, acute liver failure and intra-abdominal bleeding). In this study, the shunt traversed the tumor in 6.3% of patients. This finding is of particular concern as it may suggest that TIPS can be used without the need for traversing the tumor in most cases. In addition, a recent systematic review by Zhao et al. [17], involving 280 patients with HCC undergoing TIPS creation, also reported a high technical success rate (99%), with only 2 cases of major complications (i.e., acute liver failure and multiorgan failure). Altogether, these results suggest that both the strict selection of suitable cases and the experience of skilled TIPS operators are able to ensure severe-procedure-related complications are rare. However, from a statistical point of view, complication occurrence must be considered. 

In particular, stent dysfunction and hepatic encephalopathy (HE) are recognized as common major complications of TIPS. In a recent meta-analysis, TIPS dysfunction was defined as the need for reintervention or clinical relapse, and its pooled incidence was 23% [18]. A possible cause of the increasing incidence of shunt dysfunction might be related to bare stent placement; there is significantly lower incidence in patients with polytetrafluoroethylene (PTFE)-coated stents. In the same study, HE occurred in 29.4% of the patients during follow up. Timely detection and early treatment through close postoperative observation and regular follow-up are recommended to prevent these serious adverse events.

Another anecdotal issue related to TIPS placement in the setting of HCC remains debated: the fear of HCC progression and tumor spread, which can occur, especially in centrally located nodules, because the creation of a connection between the portal vein and the hepatic vein could provide a route for cancer cell metastasis. However, the evidence of the risk of tumor dissemination after TIPS placement is limited. In a study by Wallace et al. [9], lung metastasis was reported after 10 months in two out of nine patients with HCC receiving a TIPS. Conversely, Bettinger et al. [19] reported only 1 lung metastasis out of 40 patients after TIPS implantation. Liu et al. [15], recorded 7 out of 58 patients with metastases after TIPS insertion. Jiang et al. [20], showed no evidence of metastasis from vascular seeding whenever the TIPS shunt passed through the malignancy. However, a control group was not included in these investigations. Therefore, we cannot confirm that extrahepatic metastases are statistically associated with TIPS implantation in these patients. We must also acknowledge that this could be the result of natural tumor progression. An association between TIPS and HCC development was also hypothesized [21]. On the contrary, a couple of retrospective studies have demonstrated the safety and comparable rate of HCC post-TIPS placement [22,23]. The aforementioned studies with procedure-related complications are summarized in Table 1.

An open issue is whether TIPS can improve the overall survival (OS) rate of patients with HCC. In fact, the SPH remains unknown. For example, Yan et al. [24] showed that TIPS use may have survival benefits for these patients. This promising finding could be explained by an improvement in liver function, allowing the starting of subsequent anticancer therapy. In terms of prognostics, the latter can also be more conservative.

## 3. TIPS and Locoregional Treatments: Transarterial Chemoembolization (TACE) and Transarterial Radioembolization (TARE)

According to Barcelona Clinic Liver Cancer (BCLC) staging, the Child–Pugh score influences HCC treatment choice. In fact, the Child–Pugh score predicts the outcomes of locoregional treatment in terms of liver decompensation and death. Because decompensated liver cirrhosis is usually a contraindication to locoregional treatments, TIPS use may offer the possibility to reach the goals through the resolution of refractory ascites and, in general, of decompensated liver function [25]. However, considering the changes in venous and arterial flow before and after the TIPS procedure, the efficacy and toxicity of TACE in combination with the TIPS procedure require special attention by physicians [26]. Subsequently, several studies have been conducted to evaluate the safety and efficacy of TIPS use in combination with locoregional treatments. 

In a multicentric study, Huzheng Yan et al. [27] enrolled 68 patients with HCC and portal-hypertension-related refractory ascites. After TIPS implantation, patients were reassessed with the Child–Pugh score and evaluated for anti-HCC treatment. Of the patients, 14.7% received TACE alone, 30.9% received TACE combined with microwave ablation, and 14.7% received TACE combined with systemic therapy. The latter had a median OS of 8.7 months. Importantly, the authors estimated that TIPS use may have increased the chance of achieving locoregional treatment by at least 16.2%, explaining the improved survival rate. 

Bin Qiu et al. [14] analyzed 261 patients with HCC and portal hypertension undergoing TIPS placement together with other interventional treatments. Within this cohort, 185 patients received TACE/transarterial embolization (TAE), and 113 received percutaneous radiofrequency ablations (RFAs) alone or in combination with other treatments. No procedure-related deaths or serious complications (e.g., abdominal bleeding, hepatic failure, or distant metastasis) were recorded. Furthermore, in a retrospective study, Hai-Lin Lu et al. [28] compared the clinical outcomes and survival rate between two groups of patients, namely, those with TIPS placement who underwent locoregional treatment (TACE/TAE) and patients without TIPS placement undergoing the same procedures. The mean survival time in the TIPS group was 14 months vs. 9.9 months in the only-TACE group (*p* = 0.043). According to mRECIST criteria, there was a higher response rate to treatment of HCC patients in the TIPS group vs. those not with a TIPS (65.4% vs. 38.7%, respectively; *p* = 0.019). Furthermore, in the TIPS group, the better control of ascites and variceal bleeding was achievedd compared with the non-TIPS group (*p* = 0.045 and 0.039, respectively). The authors did not report procedure-related deaths in either group. There was no significant difference in terms of postembolization syndrome occurrence or extrahepatic metastases incidence. However, hepatic failure incidence was higher in the TIPS group vs. the other group treated only with TACE/TAE (specifically, five vs. four patients, respectively; *p* = 0.028).

Another retrospective study enrolled 50 patients undergoing TACE for HCC treatment. These patients were divided in 2 groups (25 patients with preexisting TIPS vs. 25 controls). Patients showed only one severe adverse event (namely, severe bilirubin increase) in the TIPS group, although the TIPS group had worse baseline liver function than the controls [29]. 

On the other hand, John T. Miura et al. [30] reported 25% serious adverse events after TACE in a small group of 16 HCC patients with TIPS. In detail, Clavien grade 3 or higher score postprocedure complications occurred at three separate time points. In detail, three patients developed ascites after TACE, and two patients reported liver failure; more peri-procedural complications were observed during subsequent TACE vs. previous treatments. The Clavien Classification used in this study is a method of grading postoperative complications based on the therapy used to manage them. In particular, grade 1 complications are defined as minor risk events that do not require special treatment; grade 2 events require specific pharmaceutical therapy; grade 3 events require surgical, endoscopic, or radiological interventions; grade 4 events include life-threatening complications requiring intensive care management; grade 5 corresponds to patient death [31].

In another retrospective study [10], 158 patients with comparable MELD scores (10 of them had a TIPS) were analyzed. Hepato-biliary severe adverse events after TACE procedure were recorded: they appeared to be nearly doubled in patients with TIPS (70%) vs. patients without (36%) (*p* < 0.05). Moreover, the liver transplantation rate within one year after TACE was 2.5 times higher in patients with a TIPS than in patients without it (80% vs. 32%, *p* < 0.05). However, the authors reported no significant difference at 1-year survival between the groups. 

You-Chen Kuo et al. [32] retrospectively compared patients with and without a TIPS undergoing conventional TACE for HCC treatment. The complete response rate and objective response rate were higher in the non-TIPS group (74 vs. 30% and 83 vs. 50%). The percentage of liver transplantation and the time to tumor progression between the two group were similar. Interestingly, the non-TIPS group had a significantly better OS when controlled for liver transplantation.

Specifically, only a few studies have evaluated the safety and efficacy of multihit TACE in patients with a TIPS. Ji-Won Kang et al. [33] studied 20 patients each undergoing single or multiple TACE for HCC treatment after TIPS placement. After the TACE procedure, 70% of patients showed tumor reduction, with a median survival period of 23 months. Only one patient experienced a major complication (namely, spontaneous bacterial peritonitis). From multivariate Cox regression analysis, it was shown that tumor stage only was the independent prognostic factor affecting patient survival (*p* = 0.049). In another retrospective study [34], the safety and long-term outcomes of a subsequent TACE session in 19 patients with TIPS placement were investigated. Within one month, grade 3 or 4 serious adverse events occurred in six patients. However, tumor response after multiple TACE sessions was the only predictive risk factor for mortality (OR = 4.40; *p* = 0.030; 95% CI, 1.15–16.85).

Assuming that conventional TACE (cTACE) could further reduce hepatic blood flow, which could accelerate liver functioning deterioration, Wenzhe Fan et al. [35] retrospectively compared the safety and effectiveness of this procedure in patients with drug-eluting beads (DEBs) (namely, those reported to have lower incidences of systemic adverse events and hepatotoxicity) [36] vs. those undergoing cTACE using lipiodol-based regimens with a TIPS. The incidence of adverse events, including hepatic failure within 30 days, were significantly lower in the DEB-TACE group than in the cTACE group (5.3% vs. 19.4%, *p* = 0.027). Moreover, the DEB-TACE group showed milder liver toxicity and had a better objective response rate (70.2% vs. 50.0%), median OS, and time to progression vs. the cTACE group (11.4 vs. 9.1 months, hazard ratio (HR) = 2.46, and *p* < 0.001; 6.9 vs. 5.2 months, HR = 1.47, and *p* = 0.045, respectively).

In a meta-analysis, Xi Chen et al. [37] evaluated the impact of TIPS use on the effect of TACE on patients with HCC. This study showed that the effectiveness of TACE and 1-year OS were not influenced by TIPS placement. The most common adverse event reported was hepatic failure, and the pooled hepatic failure rate was 8.8% (95% CI: 5.2% to 12.4%). Of note, the pooled hepatic failure rate increased to 12.7% (95% CI: 5.7% to 19.7%) excluding patients who received a TIPS after TACE. These results are of particular interest for physicians because they underline the importance and weight of the procedures’ order of application on the incidence of liver failure. Accordingly, a similar trend was noted in another study [38] including 79 patients undergoing TIPS before TACE. Indeed, in these patients, hepatic function recovery was difficult to reach: 13 subjects developed hepatic failure. Thus, this evidence supports performing TACE before TIPS, whenever feasible.

Looking at real-world data, we must consider possible complications and the need for the careful selection of patients who can benefit from TACE after TIPS placement. Here, Wenzhe Fan et al. [39] tried to develop a specific prognostic model. Specifically, they retrospectively evaluated 512 patients with unresectable HCC who underwent TACE after TIPS placement who were enrolled from 15 centers. Through multivariate Cox regression analysis, they evaluated the most significant prognostic factors: vascular invasion (VI), log10 (alfa-fetoprotein (AFP)), 1/creatinine, extrahepatic spread (EHS), and log10 (ALT). Through this approach, they were able to develop a new score, named VACEA (taken from the initials of VI, ALT, creatinine, EHS, and AFP), which includes these five features. According to the authors, the VACEA score is used to predict prognosis and stratify patients in four distinct risk categories. In particular, grade 1 and 2 patients showed a median OS of 25.2 and 15.1 months, respectively, indicating that they are good candidates for TACE; grade 3 patients achieved a median OS of 8.9 months; grade 4 patients had no survival benefits from TACE. For that reason, this last category of patients should be managed only with systemic therapy or palliative care.

Looking at the special categories of locoregional treatments their interaction with TIPS use, we must mention the radio-embolization procedure. Although yttrium-90 radioembolization (Y90) outperforms transarterial chemoembolization for time to progression (TTP) in HCC patients with preserved liver function [40], only a few studies have evaluated the efficacy and safety of the procedure in patient with a TIPS. Gordon et al. [41] assessed the safety and efficacy of the procedure in patients with HCC and a TIPS in a retrospective study. Thirty-nine patients were evaluated and their 30-day mortality was 0%. Grade 3+ clinical adverse events and grade 3+ hyperbilirubinemia occurred in 5% (2/39) and 0% (0/39) of patients, respectively. Regarding the procedures’ efficacy, a radiologic response was achieved in 58% (according to the WHO criteria) and 74% (according EASL criteria), respectively. Furthermore, the median TTP was 16.1 months for any cause and 27.5 months for primary index lesions. The median OS was 31.6 months and 62.9 months for censored and uncensored OLT, respectively. Indeed, the results using this procedure are not homogenous. Gabr et al. retrospectively evaluated the lung shunt fraction (LSF) prior TARE in HCC patients. The authors observed that the presence of a TIPS was associated with a significantly greater LSF vs. that in patients without a TIPS (*p* < 0.001). Thus, pretreatment with Tc-99m macroaggregated albumin (MAA) scans should be mandatory in this subgroup of patients in order to avoid the risk of radiation pneumonitis [42]. 

In conclusion, the use of TIPS generally is suitable for patients with decompensated liver disease. Moreover, liver perfusion is altered by TIPS placement. Altogether, these items raise questions as to whether locoregional treatments can constitute a safe and effective strategy. In detail, when considering which TIPS patients are good candidates for locoregional treatments, good performance status and clinical and laboratory evidence of preserved liver function (i.e., Child–Pugh A or B after TIPS) should be considered [43]. In this regard, the proposed VACEA score could be a useful tool for stratifying patients with decompensated cirrhosis undergoing TACE. Furthermore, once a patient has been screened, the super-selective DEB-TACE should be used instead of cTACE whenever available. Finally, studies analyzing the efficacy and safety of radioembolization in these patients are scarce, although first results are promising. Thus, we must wait for future comparative effectiveness studies.

## 4. TIPS Use as a Bridge to Systemic Therapy

Since 2008, the prognosis of HCC patients with BCLC stage C has improved due to the introduction of the oral multikinase inhibitor sorafenib, which has increased the OS time by 2.8 months [44]. More recently, other treatments such as lenvatinib (an inhibitor of vascular endothelial growth factor (VEGF) receptors 1–3, fibroblast growth factor (FGF) receptors 1–4, and platelet-derived growth factor (PDGF) receptor α) have proven noninferiority in terms of OS in treated vs. untreated advanced HCC patients [45]. Finally, during the last few years, a novel, more efficient breakthrough in systemic therapy emerged and is showing exciting results in terms of OS (19.2 months) [46] and PFS (6.8 months). Indeed, the combination of atezolizumab (anti-PDL1) and bevacizumab (anti-VEGF) is currently the first-line treatment for hepatocellular carcinoma [11,47], and new trials involving immune checkpoint inhibitors (anti-PD1/PD-L1 or anti-CTLA4) combined with molecularly targeted drugs are currently ongoing, producing promising preliminary results [48,49,50,51,52]. However, the administration of these promising treatments requires a Child–Pugh A status and a minimum risk of bleeding in order to avoid acute hepatic failure. Therefore, systemic therapy is highly discouraged in patients with CSPH and decompensated liver cirrhosis. In fact, the prognosis of patients with untreated advanced HCC (BCLC C or D) is poor [53]. Consequently, a high percentage of deaths is caused by upper gastrointestinal bleeding (34.1%), refractory ascites, or hepatic failure. The latter is mainly due to portal vein obstruction and thrombosis and not to liver cancer progression [54]. 

In this scenario, TIPS use has a pivotal role in the management of decompensated cirrhotic patients with advanced HCC. Although up-to-date guidelines are unclear about symptomatic portal hypertension and portal vein thrombosis treatment in advanced HCC patients, recent evidence from the literature supports the use of TIPS and sequential systemic therapy in these patients [55].

Recently, a Chinese group of researchers proposed a new flowchart for the downstaging of refractory ascites through the use of antitumor therapy. This flowchart is based on the new BCLC staging: 47.1% of this small cohort of enrolled patients received systemic therapy (namely, sorafenib, lenvatinib, or apatinib) and showed the feasibility and effectiveness of this new sequential treatment of HCC [27]. Interestingly, the modified step-by-step approach may shorten the distance between BCLC-C and BCLC-D due to CSPH and may extend survival time of patients (from 3 months to 2 years) [11].

## 5. TIPS as a Bridge to Surgery

Hepatic resection and liver transplantation are curative approaches in cirrhotic patients with HCC. However, patient eligibility is affected by tumor staging; residual liver function; and, last but not least, the presence of CSPH. The latter is significantly associated with an increased risk of postoperative liver decompensation and/or major bleeding. In fact, although CSPH (defined as HVPG > 10 mmHg and the presence of splenomegaly or platelets count < 10,000/mmc) is not considered an absolute contraindication to surgery, EASL guidelines suggest carefully selecting patients with HCC and CSPH through an accurate multidisciplinary comparison of risks vs. noninvasive approaches [43].

In detail, Boleslawski et al. [56] showed that an HVPG > 10 mmHg, perhaps measured before liver resection, strongly correlates with 90-day liver disfunction occurrence and postoperative deaths. The value of this cut-off encourages clinicians to routinely measure HVPG before liver resection. Thus, patients with CSPH should be referred to highly qualified centers for hepatology and hepato-biliary surgery [57]. Another analysis by Cortese et al. [58] showed that in a cohort of patients affected by liver cirrhosis and HCC and preserved liver function (Child–Pugh score A and no previous decompensations), the number of postoperative decompensation episodes was four-fold higher among patients with an HVPG > 10 mmHg. Of mention, this issue was also described as having acceptable percentages of mortality (<5%) and severe liver disfunction rate (<25%). Altogether, this evidence paves the road toward a surgical option in well-selected patients.

Indeed, portal hypertension and its assessment also have a role in guiding physicians regarding HCC aggressiveness and its recurrence rate after liver resection. For example, Marasco et al. [59] demonstrated that spleen stiffness (the major noninvasive parameter of HVPG in patients with CSPH [60]) is the only independent variable that correlated with tardive HCC recurrence (namely, after 3 years from liver resection). Moreover, at a molecular level, the relationship between portal hypertension and HCC development can be explained considering the onset of shear and oxidative stress due to chronic inflammation and the fibrotic distortion of sinusoids with increased levels of proinflammatory and neo-angiogenetic molecules [61]. This is followed by reduced a homeostatic autophagic phenomenon, promoting the development of more aggressive phenotypes of HCC [62]. Consequently, the portal hypertension evaluation in patients affected by cirrhosis complicated by HCC has a primary role in surgical settings. Reverter et al. [63] studied the prognostic impact of HVPG on the 90-day and 1-year outcomes of patients undergoing extrahepatic surgery. Interestingly, they determined HVPG threshold levels with progressively increased mortality risk rates. Furthermore, two reviews have demonstrated that TIPS positioning in cirrhotic patients undergoing extrahepatic surgery is safe [64,65]. Within this framework, a French group also showed that fewer ascites decompensation episodes were recorded in cirrhotic patients with CSPH receiving pre-emptive TIPS, namely, before undergoing extrahepatic surgery. However, we must take into account that mortality and severe disfunctions rates were comparable [66] among TIPS- and non-TIPS-pretreated patients.

We must also recognize the limitations in drawing conclusions from this evidence on the role of pre-emptive TIPS positioning in hepatic surgery for malignancies. A smaller number of patients are affected by cirrhosis and HCC with enough residual hepatic function to undergo a “double hit”, as represented by TIPS placement before liver resection. In a pilot study, Fares et al. [67] showed that TIPS placement before liver resection failed to improve the surgical outcome. However, it was not related to TIPS-associated complications or liver disfunctions but with a high rate of tumor disease progression, suggesting a primary role in neoplasm malignancy characteristics.

In this field of investigation, Takemura et al. [68] further highlighted the role of perioperative portal vein pressure management (e.g., endoscopic varices ligation or Hassab decongestion operation) to improve prognosis in patients undergoing hepatectomy for hepatocarcinoma. An interesting case report was described by Polacco et al. [13], realizing a surgical porto-systemic shunt in order to achieve a “salvage” hepatectomy to control HCC progression in a patient affected by decompensated cirrhosis, perhaps a candidate for a liver transplant.

Two case reports described hepatic surgery after a TIPS procedure in cirrhotic patients affected by HCC. The first patient [69] was diagnosed with one nodule of hepatocarcinoma (50 mm, segment VII) in compensated cirrhosis (Child–Pugh A5) complicated with low platelet count (<100,000/mmc): anatomic segmentectomy was realized after TIPS positioning between middle hepatic vein and left portal vein branch. The second patient with a TIPS [70], which was previously received for decompensation, underwent liver resection of intermediate HCC (11 cm) without severe complications.

TIPS use is also a valid option in cirrhotic patients affected by HCC on the waiting list for orthotopic liver transplant (OLT). Saad et al. [12] showed that TIPS use does not any technical difficulty during liver transplantation because of the intrahepatic position of the stent, which is completely removed during hepatectomy [71]. Moreover, TIPS use does not increase the hemodynamic or metabolic risk rate during anesthesia for liver transplantation [72]. For these reasons, TIPS placement can be considered a bridge therapy in patients on the waiting list for OLT in order to reduce liver decompensation episodes, improve transplant-free survival, and increase the number of successful liver transplants [73]. The data reported by Larrey et al. [74] favor this approach: eight patients with HCC undergoing TIPS positioning due to CSPH complications did not show tumor spreading or liver failure. Importantly, there was a good outcome after OLT, although patients received locoregional treatments before TIPS placement or they underwent ablations/TACE after TIPS placement.

For all these reasons, we can reasonably confirm that TIPS can be safely considered in HCC patients with CSPH on the waiting list for OLT, which should not represent a contraindication to other treatments to control HCC prior to transplant [75]. The studies investigating the role of surgery in patients with HCC who underwent TIPS positioning are summarized in Table 2.

## 6. Conclusions

TIPS placement is technically feasible and safe in HCC patients with CSPH when performed in selected cases. TIPS use may improve liver function, enabling subsequent anticancer therapies in HCC patients, potentially leading to better outcomes. The combination of TIPS use with locoregional treatments and systemic therapy shows promising results and may improve survival rates. Moreover, TIPS can be safely considered, upon multidisciplinary evaluation, in patients with CSPH and resectable HCC or candidates for OLT. Patient selection for TIPS use in HCC with CSPH should be based on a multidisciplinary approach involving hepatologists, interventional radiologists, and oncologists. Careful consideration should be given to the overall liver function, tumor characteristics, and the of other comorbidities. Experienced surgeons should perform TIPS procedures in HCC patients to minimize the risk of complications. Close monitoring and surveillance for potential tumor-related complications, such as rupture and dissemination, should be carried out following TIPS placement. Long-term studies evaluating the impact of TIPS use on OS and quality of life in HCC patients are necessary. Multicenter prospective studies are needed to provide stronger evidence of the selection criteria, safety, and efficacy of TIPS use in HCC patients in different therapeutic scenarios. These studies should include larger sample sizes, control groups, and standardized protocols to address the current knowledge gaps and provide more definitive recommendations.

## Figures and Tables

**Table 1:** Brief summary of studies with main TIPS-related complications in patients with HCC.

Study	Study Type	Number of Subjects	Child–Pugh Score	Median Follow-Up	Technical Success	Liver Failure	Tumor Rupture	Stent Disfunction	Dissemination
Qiu B et al., 2015 [14]	Retrospective	209	A 83 (39.7%) B 72 (34.4%) C 54 (25.8%)	Until death or 5 years	209 (100%)	0 (0%)	N/A	122 (58.4%)	0 (0%)
Liu L et al., 2013 [15]	Retrospective	58	A 11 (19%) B 34 (58.6%) C 13 (22.4%)	78.5 days	58 (100%)	0 (0%)	5 (8.6%)	12 (20.7%)	7 (12%, lung and abdominal lymph nodes metastasis)
Tsauo J et al., 2021 [16]	Retrospective	126	N/A	13.9 months	124 (94.4%)	1 (0.79%)	1 (0.79%)	15 (11.9% mean follow up time of 11.4 months)	3 (2.4% lung metastasis)
Bettinger, et al., 2015 [19]	Retrospective	40	A 3 (7.5%) B 29 (72.5%) C 8 (20%)	211 days	40 (100%)	2 (5%)	0 (0%)	8 (20%)	1 (2.5%)
Jiang et al., 2004 [20]	Retrospective	14	A 0 (0%) B 4 (28.6%) C 10 (71.4%)	90 days	10 (71.4%)	N/A	N/A	N/A	0 (0%, 3 months follow up)
Wallace et al., 2003 [9]	Retrospective	9 (all TIPS trough HCC)	N/A	229 days	9 (100%)	1 (11%)	N/A	3 (33.3%)	2 (22%, lung metastasis)

**Table 2:** Brief summary of studies investigating surgery options in patients with HCC who underwent TIPS positioning.

Study	Study Type	Number of Subjects	Child–Pugh Score	MELD (Mean)	Type of Surgery	BCLC	Rate of Complications
Fares et al., 2017 [67]	Retrospective	4	A5: 2 (50%) A6: 2 (50%)	8	Atypical segmentectomy: 1 Left hepatectomy: 1 Atypical resection: 1 Tumorectomy: 1	Stage A: 4 (100%)	50% (1 scare disunion, 1 persistent jaundice and ascites)
Chalret du Rieu et al., 2009 [69]	Case report	1	A5	N/A	Segmentectomy	Stage A	0%
Sliwinski et al., 2023 [70]	Case report	1	N/A	N/A	N/A	Stage A	0%
Larrey et al., 2021 [74]	Retrospective	8	A5: 1 (12.5%) A6: 2 (25%) B7: 2 (25%) B8: 2 (25%) C11: 1 (12.5%)	12,8	OLT (100%)	Stage A: 8 (100%)	0%
